# Potential niche expansion of the American mink invading a remote island free of native-predatory mammals

**DOI:** 10.1371/journal.pone.0194745

**Published:** 2018-04-04

**Authors:** Ramiro D. Crego, Jaime E. Jiménez, Ricardo Rozzi

**Affiliations:** 1 Department of Biological Sciences, University of North Texas, Denton, TX, United States of America; 2 Institute of Ecology and Biodiversity, University of Magallanes, Puerto Williams, Chile; 3 Sub-Antarctic Biocultural Conservation Program, University of North Texas, Denton, TX, United States of America; 4 Department of Philosophy and Religion, University of North Texas, Denton, TX, United States of America; Sichuan University, CHINA

## Abstract

The success of an invasive species depends in part on its niche and the new niche opportunities that such species may find in the invaded habitat. Niche opportunities can be understood as the potential provided by a community to an invasive species to expand its niche by changes in habitat use, behavior, or diet, that favors population growth, reflected in the species occupying more habitat. This may occur under a favorable combination of access to resources that can be further favored by a lack of competitors and a release from natural enemies. The American mink (*Neovison vison*) is a crepuscular/nocturnal and semi-aquatic mustelid native to North America that generally concentrates activities at <100 m from the water. It has recently established an invasive population on Navarino Island in southern Chile. Here, the mink is now the top terrestrial predator free of predators or competitors. We hypothesized that this lack of potential predators and competitors, together with a more diurnal and terrestrial prey, have resulted in the mink expanding its spatial and temporal niche on Navarino Island as compared to that in its native habitats, expressed in occupancy of sites away from water and diurnal activity. We evaluated this by using 93 randomly-chosen camera-trap stations, occupancy models and mink daily activity patterns. Models showed a dynamic occupancy with the area occupied by mink being highest during summers and lowest in spring with seasonal changes in occupancy related to distance to water sources. Mink occupied and were active at sites up to 880 m from water sources during summers. Occupancy decreased at shorter distances from water during spring, but mink were still active at up to 300 m from water. Mink were active daylong during summers, and nocturnal and crepuscular during winter and spring. These results show that compared to the native and other invaded habitats, on Navarino Island mink use more terrestrial habitats and are more diurnal during summers, suggesting a niche expansion under new niche opportunities that may enhance the negative impacts of this predator on a myriad of small native vertebrates.

## Introduction

Invasive species are one of the most important drivers of biodiversity loss and global change [1,2], leading to an extensive body of work to better understand the invasion mechanisms and consequent impacts on invaded ecosystems. Whether a species becomes invasive after it is established in a new habitat depends on how the species responds spatially and temporally to the combination of biotic (e.g., prey, competition, predators, parasites, and mutualists) and abiotic (e.g., resources and the physical environment) characteristics of the invaded habitat [3]. Therefore, invasion success depends on the niche of the introduced species and the niche opportunities that the species may find in the invaded habitat [4].

The idea of new niche opportunities can be understood as the potential provided by a community to an invasive species to expand its niche [4]. This niche expansion can be defined in terms of changes in habitat use, behavior, or diet, that favors the per-capita rate of increase of a population, reflected in the species increasing its abundance and occupying more habitat [4]. Changes then, may occur in the fundamental niche (i.e., the sum of ecological factors where a population can maintain a viable population [5]), the realized niche (i.e., the fundamental niche altered by all biological interactions [6,7]) or both. New niche opportunities arise then when species encounter a favorable combination of environmental conditions, access to resources that can be further favored by a lack of competitors, and a release from predators, diseases, and parasites [4,6–9], that allows the species to increase population size from low densities [4]. With a lack of competitors or enemies that limit population growth, the increase in population size leads to an increase in intraspecific competition that may force individuals to also occupy suboptimal habitats [10–12]. Those individuals that adapt to new conditions in those suboptimal habitats can further expand the niche of the species by an increase in among-individual variation [13].

It has been argued that many island vertebrates show niche expansion when released from constraints of mainland predators and competitors [14]. This process may explain the success of many generalist carnivores that are introduced into island ecosystems with few or no predators or competitors, further facilitated by the naivety of local prey given the lack of co-evolutionary history that allows access to more resources [15]. Empirical field studies exploring niche expansion of an invasive predator, however, are still scarce. In this study, we assess the hypotheses of spatial and temporal niche expansion of an invasive opportunistic predator, the American mink (*Neovison vison*; hereafter “mink”), which established as a top terrestrial predator having no competition with other mammalian carnivores or predators on an island ecosystem at the southernmost forest of the world.

Native to most of the North American continent, the mink is a solitary mustelid, generalist carnivore, territorial, and of semi-aquatic and crepuscular and nocturnal habits [16]. It generally prefers one-dimensional habitats associated with riparian areas following freshwater ecosystems such as rivers and pond coasts, and marine coasts, with most of the activity concentrated <100 m from the water [16–19]. Near or in water bodies and during crepuscular and nocturnal hours mink prey on fish, crayfish, amphibians, birds, and mammals [16], resting during day hours in den sites [16,17]. The mink has an extended history of successful invasions across the world [17]. In the 1930s, the mink was brought to southern South America to establish fur farms in different locations along the Argentinean and Chilean Patagonian Andes [20]. The mink was introduced into Tierra del Fuego Island, Argentina, through accidental and deliberate releases from the fur farms [20], and it was first documented on Navarino Island, Chile, in 2001 [21].

Since the arrival to Navarino Island, the mink population expanded across most of the marine coastline and freshwater systems [22] with negative consequences for the native biota [23,24]. The mink expansion may have been favored by the lack of other mammalian competitors and the lack of potential predators [22]. Additionally, and given a lack of some of the natural food sources found in its native range, including freshwater fish, crayfish, and amphibians [16], on this island mainland birds and rodents became important components of mink’s diet [24,25]. The consumption of rodents and birds is more important for mink inhabiting inland territories (96% of ingested biomass) as compared to the marine coastline, where mink rely mainly on marine fish (72% of ingested biomass) [25]. Therefore, we hypothesized that the lack of potential predators and competitors, together with a more diurnal and terrestrial prey given the low abundance or general lack of freshwater prey, have resulted in the mink expanding its spatial niche (expressed in changes in occupancy) and temporal niche (changes in activity patterns) on Navarino Island, as compared to native habitats where mink restricts most of its activity near water (<100 m) and is mostly crepuscular and nocturnal. We predicted a spatial niche expansion from semi-aquatic habitats along rivers and coastlines (its natural niche) that will be expressed in mink occupying the marine coastline, freshwater systems, but also more terrestrial habitats away from water sources. In addition, we predicted a temporal niche expansion expressed as an increase in mink diurnal activity as its main prey, birds and rodents, present also diurnal activity. We sought to evaluate our predictions by using camera traps to investigate i) the habitat variables that determine mink patterns of seasonal occupancy dynamics in the marine coastal-terrestrial environment by fitting a Bayesian hierarchical multi-season occupancy model; ii) the relationship of mink occupancy and activity in terrestrial habitats at different distances from freshwater sources by fitting single season Bayesian occupancy model and determining mink detection rate; and iii) the seasonal daily mink activity patterns. We used occupancy instead of abundance given the difficulties to identify mink individuals [26].

## Methods

### Study area

**Fig 1:**
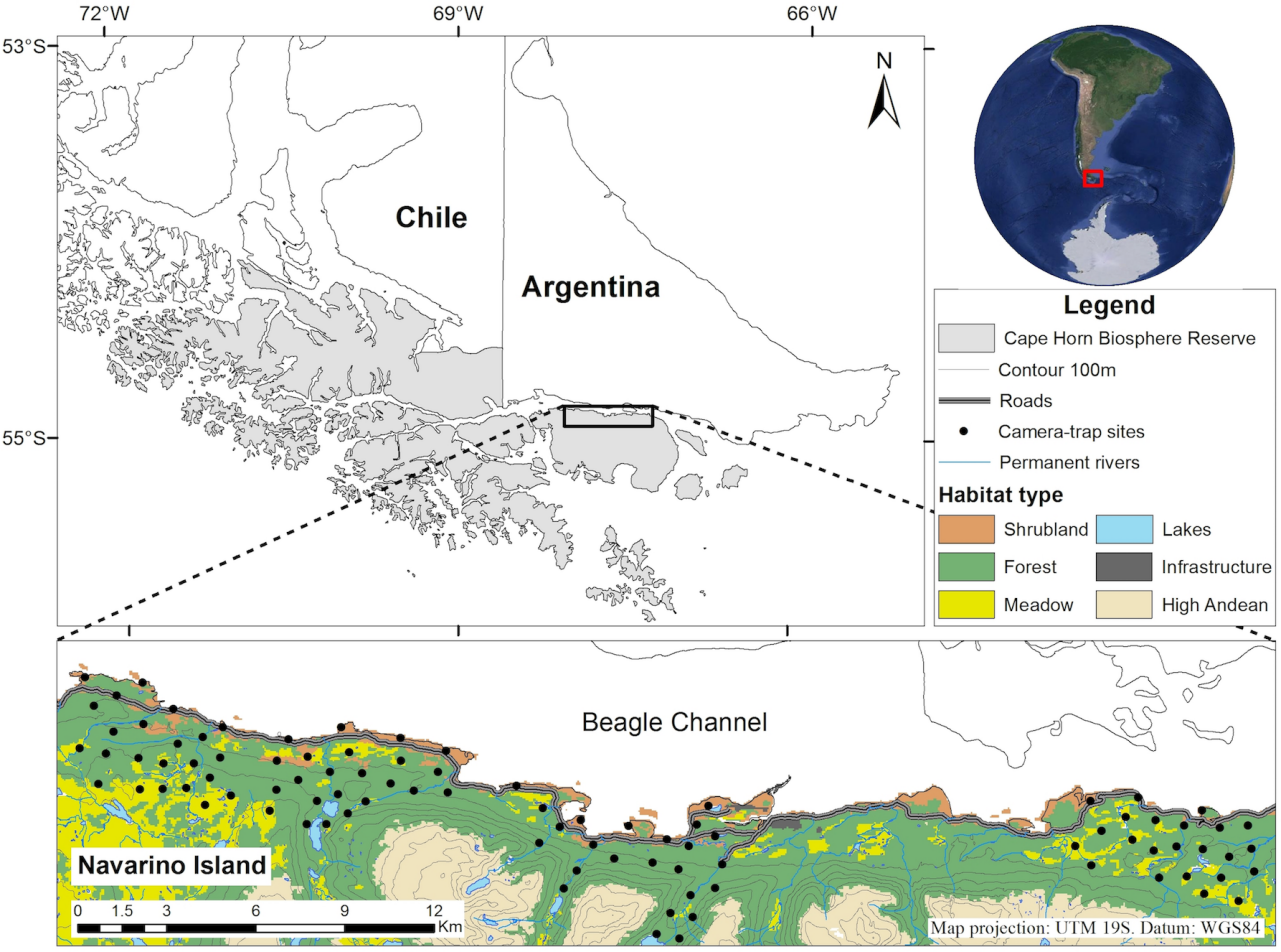
Study area location and study design. Location of 93 camera-trap stations used to study occupancy and temporal activity of the American mink on the northern slope of Navarino Island, within the Cape Horn Biosphere Reserve, southern Chile.

We conducted this study on the northern slope of Navarino Island (55°S, 68°W, ca. 2500 km^2^), located south of Tierra del Fuego, within the Cape Horn Biosphere Reserve (CHBR; Fig 1). The CHBR protects the pristine Magellanic Sub-Antarctic ecoregion [27]. The climate has a strong oceanic influence, with a mean annual temperature of 6° ± 5°C and uniform precipitation with an annual average of 467 mm [28]. The topography presents a mountain landscape, encompassing four distinct habitat types: shrublands along the marine coasts, deciduous and evergreen forests on the slopes up to 500 m in altitude, moorlands including meadows and peatlands, and high-Andean vegetation above the tree line [28] (Fig 1). Many of the current meadows are the result of invasive American beaver (*Castor canadensis*) activity that has transformed part of the native forest into meadows [28].

The vertebrate fauna on Navarino Island is restricted to birds, mammals, and fish. Amphibians and reptiles are absent [28]. Birds are the most abundant group with approximately 34 species from 20 families, with 18 species being Passeriformes [29]. Terrestrial mammal species are scarce. There are only two species of native rodents, two species of native bats, and one native ungulate [30]. In addition to the mink, American beavers, muskrats (*Ondatra zibethicus*), and house mice (*Mus musculus*) were introduced on Navarino [30]. Also cows, horses, pigs, dogs, and cats can be found freely roaming in many sectors of the island [30]. In the freshwater systems, freshwater crabs are absent and only three species of native fish and two introduced salmonids can be found in low abundances, with a mean abundance for all species being <0.04 fish/m^2^ [31]. In contrast, the marine fish and crustacean fauna in the Beagle Channel is rich and abundant with more than 50 species [32]. On Navarino Island, mink lack mammalian competitors, and the only potential predators are large birds of prey and feral dogs. Owls and eagles occur in low densities and are rarely documented [30], thus their effect on mink can be neglected. Mink rests have not been documented in dog’s diet on this island [33].

### Camera trap data collection

Occupancy model assumptions include that the species is unequivocally identified, that detection histories are independent, and that there is no change in the occupancy status during a season [26]. We developed a protocol to operate 93 random selected stations during the following four periods: Feb.-Mar. (late summer) 2014 and 2015, Jun.-Jul. (winter) 2014, and Sept.-Oct. (spring; breeding season) 2014 (Fig 1). Random locations were defined within four previously identified areas of 16 to 19 km^2^ that were accessible (i.e., public or private lands from which access permission was granted) and representative of the three habitat types that mink use in the area: costal shrublands, meadows, and forests (Fig 1). We placed one camera trap (Bushnell Trophy Cam: Bushnell Corp., Overland Park, KS, USA) at each station. However, harsh weather conditions during winter 2014 impeded us to access all sites, resulting in only 47 stations monitored during this season. We designed the study attempting to balance out minimal distance between sites with limitations of terrain accessibility. The 93 random locations resulted in a mean distance of 809 m between cameras on inland habitats (forests and meadows; range = 720–1,053 m) and 1,978 m (range = 1,363–2,782 m) along the marine coastal habitat (costal shrublands). For each season, cameras were active 24 h/day for 20 days. This was divided in four sampling periods of 5 days’ intervals for which mink was either detected or not, to construct the capture history needed for the occupancy models. We lack information on mink home range for the island although five individuals were identified from pictures given a characteristic brown or grey fur coloration (different from the more common black coloration). In none of these five cases, we detected the same individual in neighboring cameras during the sampling period. This suggested that the distance between cameras and the 20 days was appropriate to comply with the closure and independence assumptions of occupancy models at the time of obtaining a balanced history of capture and non-capture events.

To ensure that mink would pass through the detection zone of the camera trap and to increase the probability of detection, we used canned fish as bait [34]. Given the difficulty to access most of the sites to rebait cameras, we placed canned fish inside a punctured can that was secured to the ground, making it impossible for a mink or scavengers to remove neither the bait nor the can. The continuous presence of bait ensured that the probability of attracting an individual was not highly biased toward the first days in detriment of later days (S1 Appendix). Cameras were placed at heights of 30–40 cm from the ground and at 2–4 m from the bait. We set each camera to trigger 3 pictures per detection with a delay time of 60 seconds. As we had 48 cameras available, we ran the 93 sites in two sequential groups.

### Habitat co-variates

Previous studies documented that mink prefer to move close to water sources, through woody vegetation and complex ground structures where they can hide, avoiding open areas and high slopes [17,22,35,36]. Therefore, we first obtained coastline, permanent rivers, lakes, and ponds geographical information for the study area from the Cape Horn Biosphere Reserve database (GIS Laboratory of Omora Foundation and Universidad de Magallanes). We also mapped beaver ponds using Google Earth imagery from 2014 and 2015 with the historical imagery function (version 7.1, Google Inc., Mountain View, CA, USA). We then calculated two covariates: the minimal lineal distance to the marine coast and the minimal lineal distance to any water body (marine or fresh water) from each camera station. Second, we calculated elevation and slope using a 30-m spatial resolution Digital Elevation Model. Finally, at each camera station we used 10-m transect along each of the four main cardinal directions and centered at each camera location, to estimate percentage of cover and height of different ground components (bare soil, litter, grass, herbaceous, trunks, shrub, and seedling) using the point-intercept method (data recorded every 50 cm). We then calculated the percentage cover of all ground components that were taller than 10 cm (ground cover), understanding that ground components above 10 cm would allow mink to cover and hide. We used ArcGIS 10 (ESRI, Redlands, CA, USA) for all geospatial work. All covariates were standardized to a mean of 0 and standard deviation of 1 for the analyses [37]. Elevation and distance to the coast were highly correlated (*rho* = 0.87); therefore, we avoided using them together in the same model structure.

### Dynamic occupancy modelling

We examined the relationships of mink site occupancy dynamics with habitat co-variates by fitting a Bayesian hierarchical multi-season occupancy model [38]. Site occupancy can be formulated as a hierarchical state-space model that links two binary regression models: one that accounts for the ecological processes that describes occupancy of sites and a second that accounts for imperfect detection [26,38]. We considered the history of capture obtained from camera traps of *i* = 1,2,…,93 sites, over *t* = 1, 2, 3 periods of time (seasons). We did not include winter for this analysis given that we could not access all sites to set cameras and measure vegetation. The model involves four probability parameters: occupancy in the first season Ψ_1_, colonization *γ* (probability that site *i* is occupied in season *t* given it was not occupied in season *t—*1); extinction *e* (probability that site *i* is not occupied in season *t* given it was occupied in season *t—*1); and detectability *p* [26,38].

Let z(*i*,*t*) denote the true occupancy status of site *i* for season one, such that z(*i*,1) = 1 if at least one mink occupied that site during season 1, and z(*i*,1) = 0 if the site was not occupied [26,38]. The initial occupancy at a site *i* can be modelled as the outcome of a Bernoulli random variable:

$$z(i,1)∼Bern(\Psi_{1})$$

and for the following seasons,

z(i,t)|z(i,t−1)∼Bern(z(i,t−1)*[1−e(t−1)]+[1−z(i,t−1)]*γ(t−1)

Thus, the probability that a site *i* is occupied at time *t* is then the sum of the product of the probability of persistence (or 1—extinction) and the probability of occupancy at time *t*-1, plus the product of the probability of colonization and the probability that the site was not occupied at time *t*-1[38]. To account for detectability, we modelled the observed data for the four sampling periods conditional on the occupancy latent process and as a function of detection probability [38]. We used the logit-link function to incorporate covariates in the model [38].

We sequentially modeled detection probability, occupancy, colonization, and extinction. For the first stage, we modeled detectability, while keeping occupancy, colonization, and extinction constant. We modeled the effect of ground cover and/or the distance to any water source on detection probability of mink. We then fit models of initial occupancy with increasing complexity in terms of elevation, slope, distance to any water source, and habitat type, while keeping colonization and extinction constant.

We finally modeled colonization and extinction. Colonization and extinction may depend on the state condition of neighboring sites. To test for the influence of the spatial structure on the dynamic parameters of extinction and colonization, we incorporated the auto-covariate *D* [39]. If *N*_*i*_ represents the set of sites that are neighbors of site *i*, and n_*i*_ is the number of neighbors of site *i*, then we can define a spatiotemporal auto-covariate *D*_*i*,*t*_ as:

$$D_{i,t}=\frac{1}{n_{i}}\sum_{j\in N_{i}}z_{j,t}$$

We allowed colonization and extinction to depend on the status of this auto-covariate and check for any effect. We also tested for the effects of habitat type and distance to the coast on colonization and extinction probability given that mink abundance on the marine coast is higher than in inland rivers and mink can be expanding from the coast [22]. Finally, we tested for the effect of the distance to all water sources (fresh water and the marine coast) to test if mink are expanding to terrestrial sites from the marine coast and freshwater systems. We also included quadratic effects, understanding that mid values of distance to the coast or all water may be more influential.

We selected the final model by performing a forward sequential model selection procedure based on the Deviance Information Criterion (DIC) [40], our understanding of the system, and the posterior distribution of key parameters. Thus, at each modeling stage, we compared the null model with models that incorporated the different co-variates. The idea was to compare models with increasing level of complexity to the model with natural variability. If more than one covariate resulted informative (lower DIC than the null model), we then continue building a more complex model with the forward stepwise procedure. We then selected the model with the lowest DIC. However, and understanding that DIC values for hierarchical models are suggestive rather than definitive, we selected models with close DIC values, but not the lowest, if the estimated model parameters were biologically equitable [41].

### Inland occupancy

On Navarino Island, mink have plenty of prey opportunities along the marine coastline due to the rich fauna of the Beagle Channel [32]. In this habitat, almost 80% of the diet is composed by marine fish and crustaceans [25]. Given the limited availability of prey in the freshwater systems of Navarino Island, it is the mink inhabiting in inland territories that may expand the niche to occupy more terrestrial habitats away from the rivers, streams, and ponds. To differentiate mink on the marine coast from mink inhabiting inland territories that may be using more habitats away from freshwater systems we fit Bayesian hierarchical single-season occupancy models [38] for each season independently, including winter, using distance to fresh water as a covariate. We used the 73 sites located in inland forests and meadows for analysis (37 sites for winter), excluding sites located along the marine coast and sites that were closer to the marine coast than to fresh water. We modelled detectability based on the most parsimonious detectability structure identified on the multi-season occupancy analysis.

To understand how frequently mink were detected—complementing the presence-absence information of the occupancy model—we also estimated relative detection rate as a function of distance from fresh water. We first divided distance in segments of 50 m, to later estimate detection rate per segment as the number of mink detections per 100 trap nights (>60 min between detections).

### Model implementation

We implemented models using program JAGS [42], through package R2jags in R programing language [43]. We used 3 chains of Markov chain Monte Carlo (MCMC) to find 100,000 posterior distribution of the parameters of interest after a 20,000 burn-in period. In all models, we used non-informative priors. We evaluated model convergence using the Gelman-Rubin diagnostic, which is near 1 for each parameter when convergence is reached [44].

### Temporal activity patterns

To evaluate temporal niche expansion of mink we estimated daily activity patterns (i.e. probability of a picture being taken at any particular time of the day) using a kernel density analysis [45]. We extracted day and time from each picture (>60 min between detections). If the camera captured two or more individuals in one photograph we treated the event as one-time data point. We defined four daily periods as dawn (two hours before and after sunrise), day (two hours after sunrise and before sunset), dusk (two hours before and after sunset), and night (two hours after sunset and before sunrise) [46]. Day-specific sunrise and sunset hours were obtained from a GPS unit in the field. We determined sunrise and sunset time as the mean time for the days in which cameras were operative during each season.

We used the non-parametric kernel density estimation to calculate the proportion of probability density distribution for each daily period [45] to then examine whether mink selected, avoided, or used dawn, day, dusk, or night as expected given the proportion of time available for each period and for each season. This selection ratio (proportion of use/proportion available) indicates the level of preference given availability, with values >1 indicating that the time period is selected and values <1 indicating that it is avoided, being significantly different from 1 if the confidence interval for the selection ratio does not contain the value 1 [47]. As most mink cannot be individualized from the pictures, we used the design I selection function, estimating selection at the population level [47], and recognizing that pseudoreplication may be present. We used overlap and adehabitatHS packages within R programing language [43] for the analysis.

## Results

### Monitoring efforts

**Fig 2:**
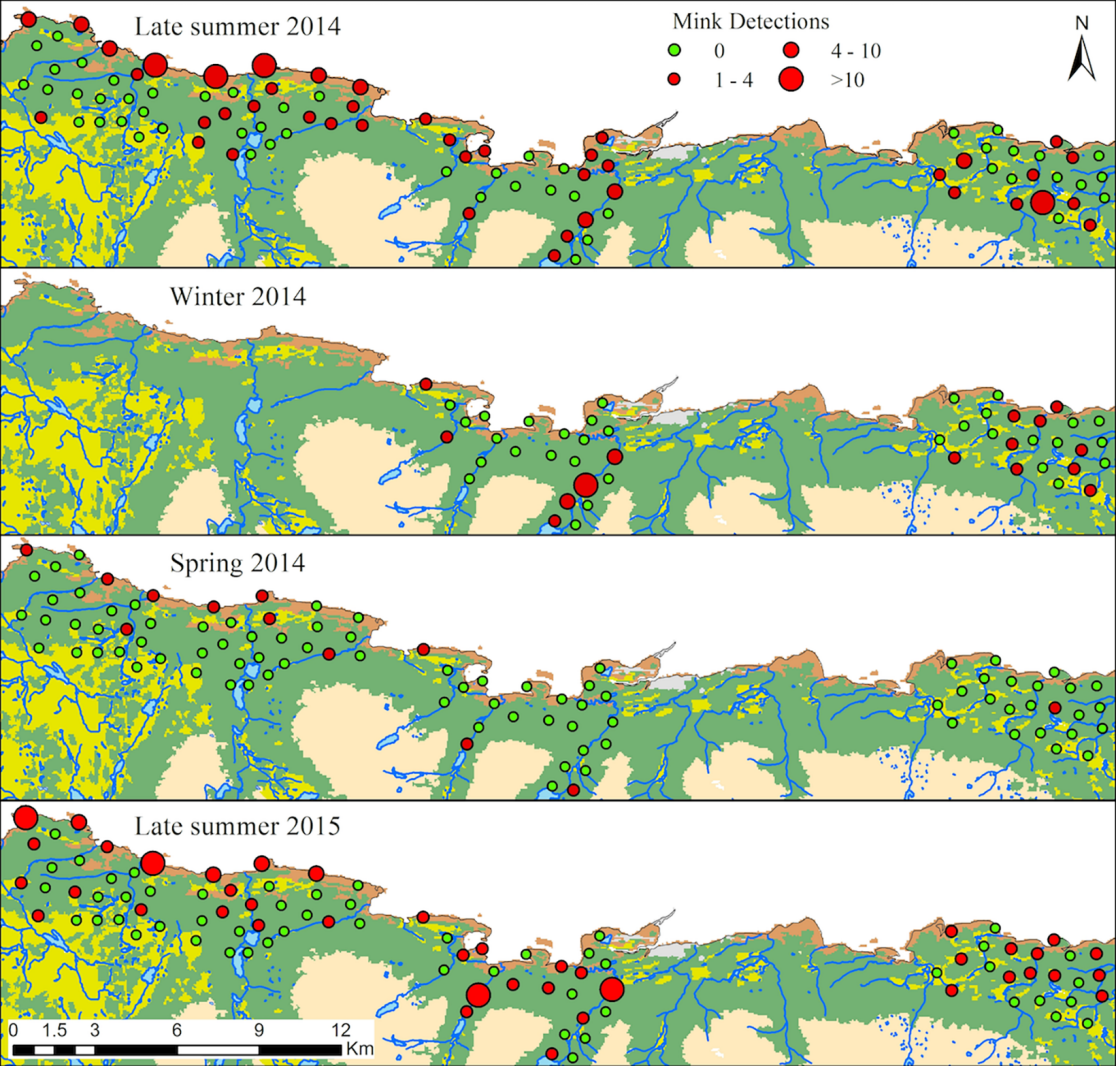
Camera trap results on Navarino Island, Chile. Figure shows mink detections during four consecutive seasons: late summer 2014, winter 2014, spring 2014, and late summer 2015. Detections are considered to be >60 min separated at each camera.

Cameras operated for 1,802 trap nights during summer 2014, 940 during winter 2014, and 1,840 during spring 2014 and summer 2015. Capture rate was 10.71 detections/100 trap nights for summer 2014. It dropped to 4.89 during winter and dropped even further to 1.14 during spring. Capture rate increased to 7.45 during summer 2015 (Fig 2).

### Dynamic habitat occupancy

**Fig 3:**
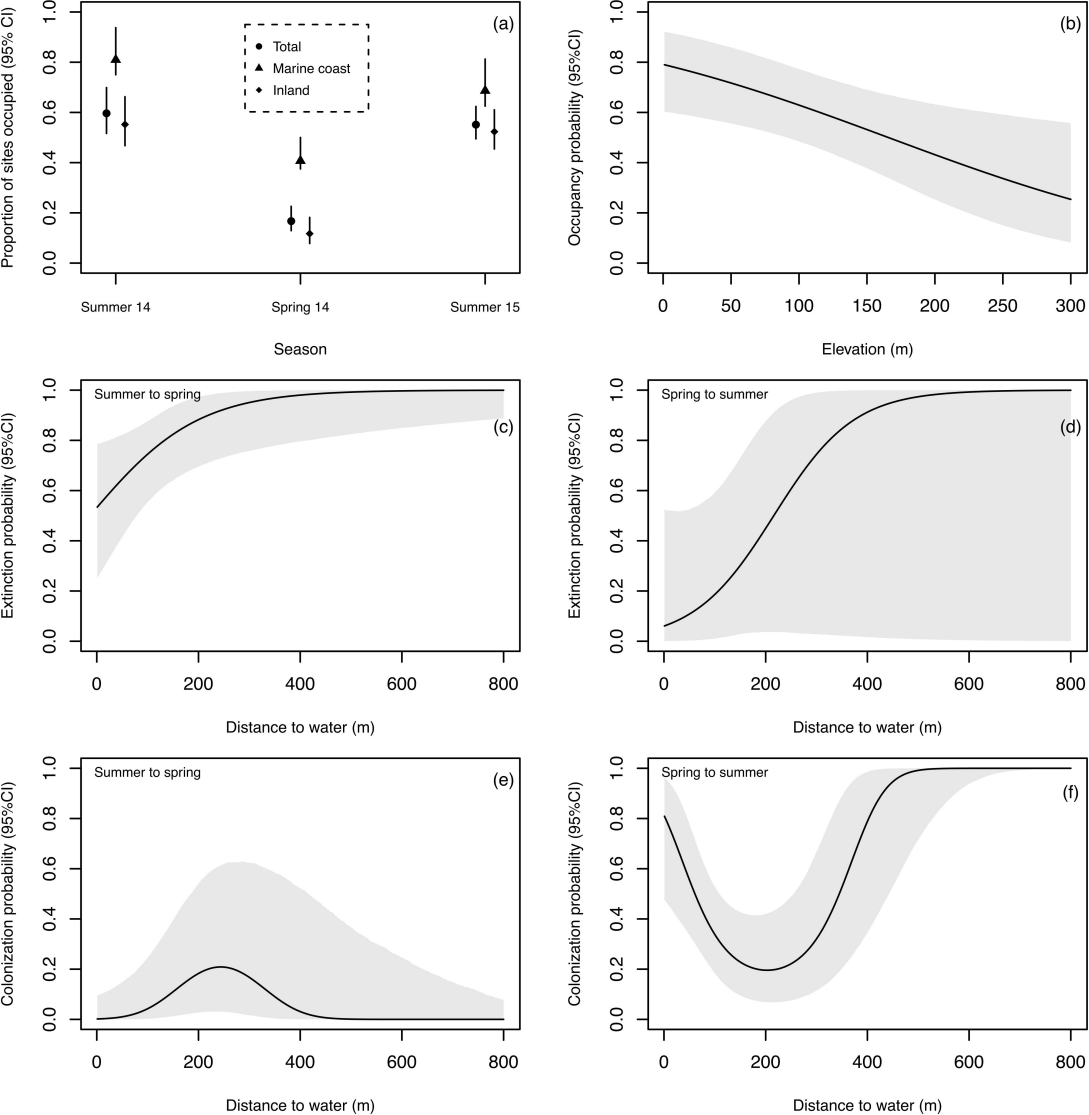
Bayesian dynamic occupancy model results. Proportion of area occupied by season (total, marine coastal habitats, and inland habitats; a), occupancy probability as a function of elevation (b), colonization and extinction probability as a function of distance from water sources (c,d,e,f) with 95% credible intervals of the American mink on Navarino Island, Chile, based on the posterior distribution of a Bayesian hierarchical multi-season occupancy model, for summer and spring 2014, and summer 2015.

Based on the posterior distribution of the most parsimonious model, mink detection probability was highly affected by distance to all water sources (marine and fresh water) and by ground cover (S1 Table). Detection was higher at closer distances from water sources and at higher ground cover (S1 Fig). The proportion of sites occupied was 59.6% during summer 2014, decreased to 16.7% during spring, and increased again to 55.2% during summer 2015 (Fig 3A). The proportion of sites occupied was higher for marine coastal habitats as compared to inland habitats (forests and meadows) for the three seasons (Fig 3A). Occupancy was affected by elevation, being higher at the sea level (closer to the marine coast) and lower at high elevations (Fig 3B). Distance to all water sources, slope, and habitat type did not result in informative co-variates to explain occupancy (S1 Table).

Neither colonization probability nor extinction probability were affected by the auto-covariate *D* (Colonization Summer 2014: -0.25 [95% Credible Intervals: -5.30 to 4.03]; Colonization Spring 2014: -0.37 [-4.13 to 3.40]; Extinction Summer 2014: -1.11 [-4.91 to 1.99]; Extinction Spring 2014: -0.37 [-5.68 to 4.59]). These models did not improve the model fit either (S1 Table). Colonization and extinction were affected by distance to any water source. From summer to spring, extinction probability was higher for sites away from water (Fig 3C) and colonization probability was low at all distances from water sources (Fig 3E). From spring to summer, extinction probability increased with distance from water (Fig 3D), however, it was uninformative with parameter value intercepting zero (S2 Fig). Nevertheless, colonization probability from spring to summer had a quadratic response. Sites with higher probability of colonization were close or far away from water, with a decrease in probability for intermediate distances (Fig 3F).

### Inland habitat occupancy

**Fig 4:**
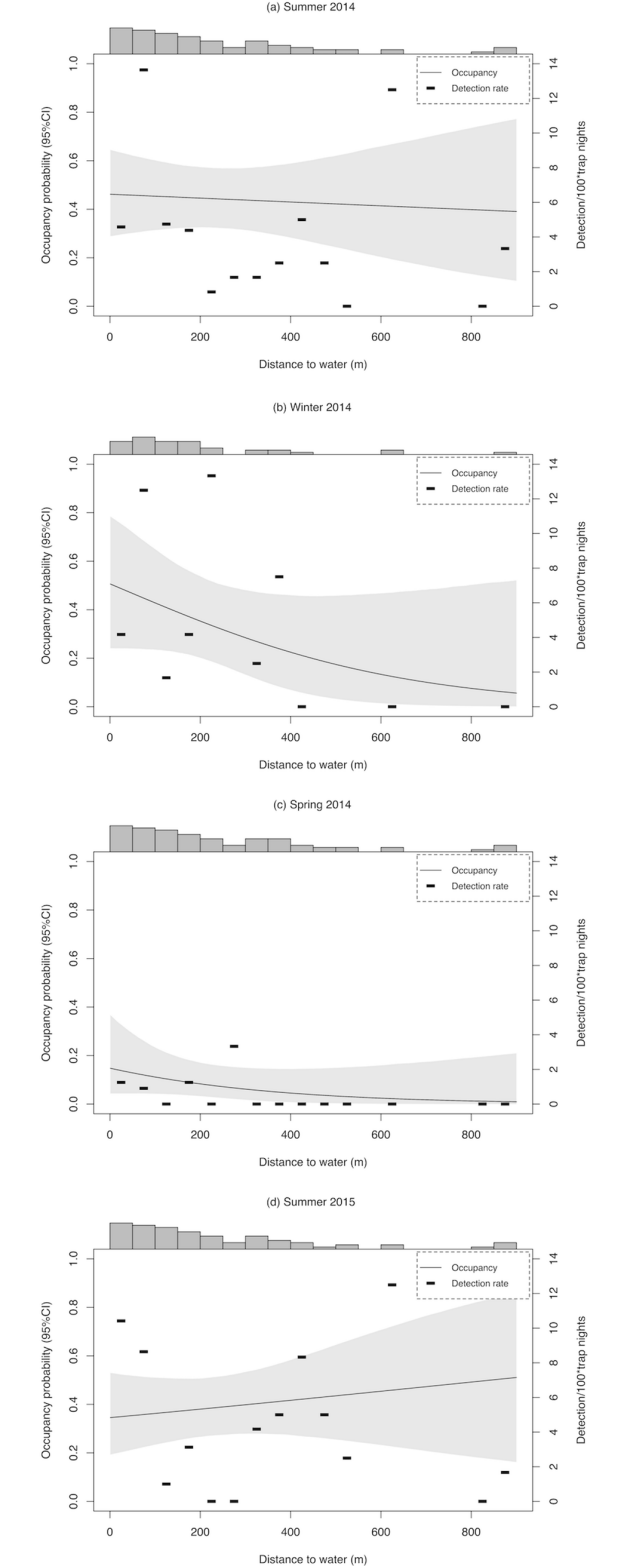
Bayesian single-season occupancy model and detection rate results. The figure shows occupancy probability as a function of distance from fresh water (95% credible intervals) based on the posterior distribution of Bayesian hierarchical single-season occupancy models of the American mink on Navarino Island, Chile, for summer, winter, and spring 2014, and summer 2015 (gray line). Detection rate measure as the number of mink detections per 100-trap nights (>60 min between detections) per segments of 50 meters of distance to fresh water is also shown (black slashes). The bars at the top of each plot represent the number of trap nights per 50 m segment.

Based on the posterior distribution of the single-season occupancy models that incorporated information from cameras placed inland, the probability of occupancy was constant at different distances from freshwater sources during the summers. In contrast, it presented a negative relationship with distance to freshwater sources during winter and spring (Fig 4). In all cases, 95% credible intervals for the parameter distance to fresh water overlapped zero, although for winter and spring the overlap was minimal (S3 Fig).

Detection rate for mink per 50 m segment followed a similar pattern than the one found for occupancy. Mink activity was detected up to 880 m from freshwater sources with no clear visual relationship with it for both summers, 2014 and 2015. Activity was restricted to sites <400 m from freshwater sources during winter and to <300 m from freshwater sources during the spring (Fig 4).

### Temporal activity patterns

**Fig 5:**
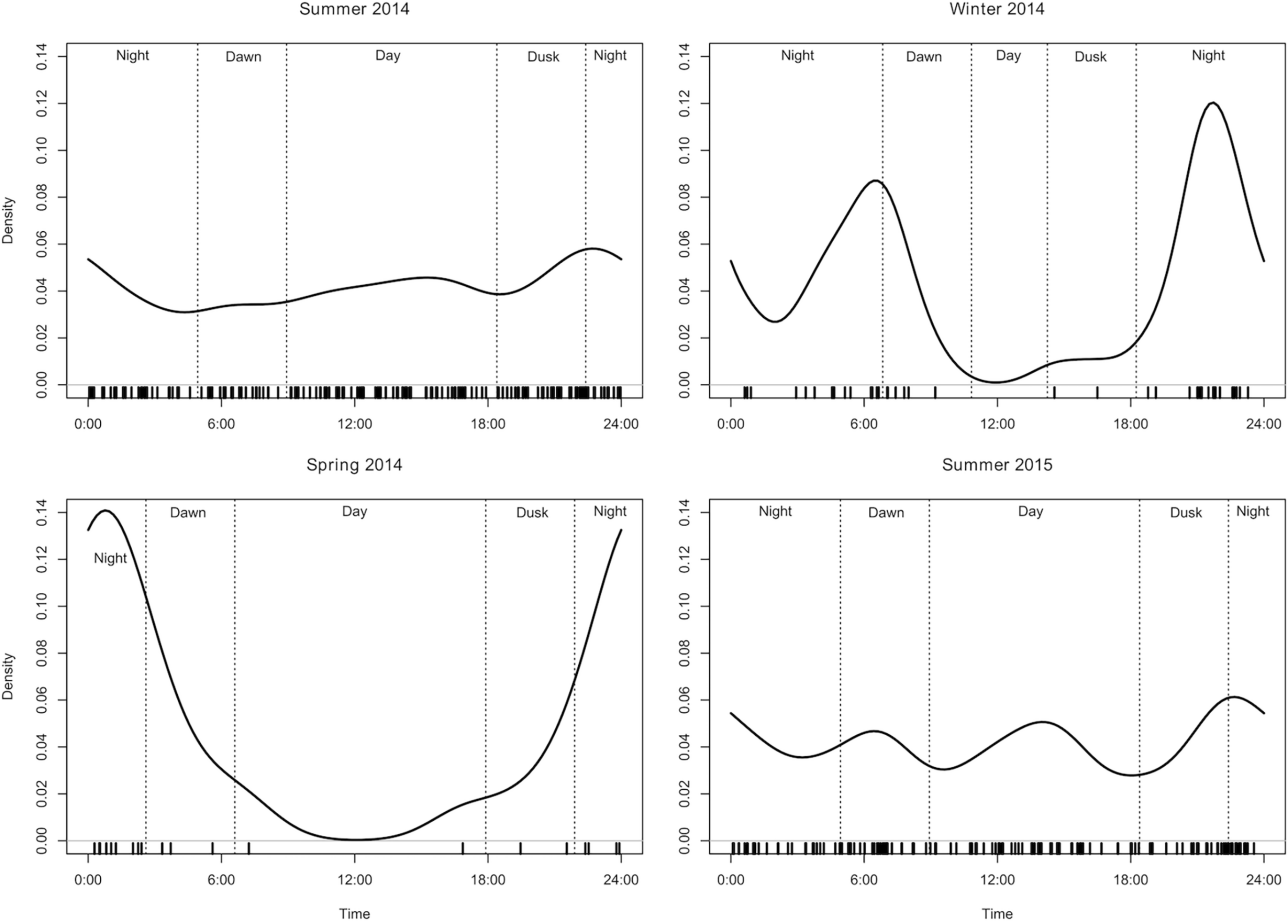
American mink daily activity patterns. These figures show the temporal activity patterns estimated as kernel density of the American mink for four seasons (summers 2014 and 2015, winter 2014, and spring 2014) on Navarino Island, Chile. Black bars at the bottom of each figure indicate mink detections (>60 min between detections).

Mink presented marked different activity patterns among summers, winter, and spring. Activity patterns were similar between summer 2014 and summer 2015 and evenly distributed along the day (Fig 5). Thus, mink were active at dawn, day, dusk, and night as expected for the time available, without selecting for any daily period (Selection ratio [Bonferroni confidence intervals] Summer 2014: dusk = 1.13 [0.61–1.64]; night = 1.02 [0.66–1.37]; day = 1.01 [0.74–1.27]; down = 0.81 [0.33–1.28]; Summer 2015: night = 1.08 [0.69–1.46]; dusk = 1.02 [0.50–1.53]; dawn = 1.01 [0.51–1.50]; day = 0.93 [0.66–1.19]). However, during winter and spring, mink concentrated activity at crepuscular and nocturnal periods of the day (Fig 5). During winter, mink selected night, used dawn as expected, and avoided dusk and day (Selection ratio [Bonferroni confidence intervals]: night = 1.51 [1.35–1.66]; dawn = 0.88 [0.40–1.35]; dusk = 0.28 [0–0.57]; day = 0.08 [0–0.23]). During spring, mink selected night, used dawn and dusk as expected, and avoided day (Selection ratio [Bonferroni confidence intervals]: night = 2.73 [2.17–3.29]; dawn = 1.36 [0.8–1.92]; dusk = 0.82 [0.34–1.29]; day = 0.22 [0.08–0.35]).

## Discussion

Our study provides evidence to partially support the hypothesis of spatial and temporal niche expansion under new niche opportunities for the American mink introduced into an island ecosystem where the mink lacks natural predators or competitors and has new prey resources. As predicted, our results show that the semi-aquatic crepuscular mink is occupying more terrestrial habitats and having more diurnal habits on Navarino Island. However, such patterns varied seasonally. The proportion of sites occupied by mink was high during summer, with occupancy being independent from distance to water and mink presence recorded at up to 880 m from water sources. Proportion of sites occupied decreased during the spring, with high extinction probability for sites away from water. However, colonization probability of sites increased from spring to summer, increasing also the proportion of sites occupied at higher distances from water. When considering only cameras placed in inland habitats isolating the effect of cameras on the marine coast, we found mink occupying terrestrial habitats independently of the distance from water during summers. During winter and spring, mink occupied sites up to 400 and 300 m from fresh water respectively. Mink were also active throughout the day during summers and showed a crepuscular and nocturnal activity during winter and spring.

Mink occupancy of terrestrial habitats up to 300 m from water yearlong, but up to 880 m in summer, suggests a spatial niche expansion from areas near water sources toward terrestrial habitats. This represents a marked difference from other studies on the semi-aquatic mink in the native range, but also from invaded ecosystems. For instance, in the native ecosystems of the U.S., mink activity was documented >100 m from water sources only 14% of the time yearlong [19]. In Europe, mink activity has been documented at distances of <50 m from rivers yearlong [36,48,49]. On Navarino Island, in contrast, we found that 43% of a total of 200 mink detections across all seasons in inland habitats occurred at >100 m from freshwater sources. Mink have been shown to shift trophic niche to more terrestrial prey when exposed to competitors such as otters and polecats [50–52], but this has not been linked to a shift towards terrestrial habitats away from water sources, probably as mink may still hunt in riparian areas close to streams.

We found that mink occupancy on Navarino Island was more stable and higher across seasons in the marine habitat than in inland habitats. The high occupancy in the marine coast is in line with the higher abundance of mink reported on the marine coast of Navarino [22] and the preference for marine coastal habitat by mink reported in Scotland [53] compared to inland rivers and ponds. The habitat a species occupies is an important dimension of a species niche [5], and when the species is released from a competitor or a predator, the realized niche can expand, allowing an increase in abundance and occupying more habitat [4,6–9]. Additionally, organisms tend in general to first occupy optimal habitats, and as competition increases and per capita habitat quality decreases, some individuals expand their habitat use to also occupy suboptimal ones [10–12]. While the presence of competitors has been shown to reduce the abundance of mink in Europe [50,54], the absence of them may favor population growth intensifying intraspecific competition. This can be the case on the marine coast of Navarino Island where mink prey on abundant fish and crustaceans along the marine coastal habitat [25]. Marine coastal habitats likely represent the optimal habitat for mink where intraspecific competition may force some mink to disperse and occupy inland territories, explaining the dynamic occupancy we observed at inland habitats (Fig 3A). This may resemble a source-sink dynamic that has been recently suggested for mink inhabiting steppe-arid environments of continental Patagonia [55].

Inland habitats on Navarino Island lack common freshwater prey such as fish and crabs, and they prey more frequently on muskrats, representing 55% of the biomass consumed during the year [25]. The remaining of the diet is composed mainly by small rodents and passerine birds [25]. However, many bird species emigrate during winter, forcing mink to concentrate predation on mammals during this season, especially on muskrats [25]. In the inland forests and meadows, we found mink occupancy to be more dynamic. The area occupied was lower during the spring as compared to summer, and in inland habitats occupancy was restricted at <300 m from water for winter as compared with summer when mink was detected up to 880 m from fresh water. It has been shown that mink foraging and travelling activities tend to decrease during cold months, with animals spending most of the time active or inactive inside or near their dens [56,57]. This may explain in part the lower capture rate and lower proportion of habitat occupied during the spring compared to the summer. However, muskrats, the main prey in inland habitats during the cold months, are highly associated with streams and beaver dams [25]. Therefore, it is reasonable to expect that the mink will be more likely to occupy habitats that are closer to freshwater sources during this time of the year, explaining also the higher probability of extinction of sites at higher distances from water towards the spring.

Our results partially support the prediction of temporal niche expansion on Navarino Island. Mink were active throughout the day during summers, presenting a temporal niche expansion, and were crepuscular and nocturnal during winter and springs, similar to the native range were mink range are mostly crepuscular and nocturnal [16]. Our results agree with recent studies in invaded habitats that showed similar increases in mink’s diurnal activity [48,50,58], generally associated with prey activity patterns [59] or competition with otters [50,60]. In the absence of otters, activity patterns of native small rodents and birds that are an important component on mink diet on Navarino Island during summers, may explain mink temporal niche expansion [24,61].

Overall, we argue that the absence of predators and competitors and the presence of more terrestrial and diurnal prey during summers (small rodents and birds) are the mechanisms explaining the occupancy of terrestrial habitats, the dynamics of occupancy at high distances from water, and the summer diurnal activity of mink. The lack of predators or competitors, together with the predator-prey interaction with small rodents and birds may represent the niche opportunities provided by the native community of Navarino Island to the invasive mink to expand its niche from being crepuscular and nocturnal and showing activity near water in its native range to be more diurnal and to occupy also terrestrial habitats away from water. However, further studies assessing terrestrial occupancy, and ideally abundances, in the native or other invaded habitats with a different ensemble of potential competitors, predators, and prey composition should be conducted to contrast and confirm this hypothesis and preliminary evidence.

The niche is defined as the set of environmental condition where a viable population can exist, and therefore, the inland territories far away from water sources may be occupied by transient individuals that may not be reproducing (e.g., male juveniles [55]), and then living outside the niche of the population [5]. However, the ecological niche defines the environmental context within which adaptive evolution occurs and niches themselves can evolve [5]. Evolutionary phenotypical changes, such as behavior flexibility in plastic species, can allow invasive species to adapt to suboptimal habitats [62] and lead to ecological disruptions that are more severe than previously predicted based on the ecology of the species in its native habitat [63]. For instance, the nocturnal brown tree snake (*Boitga irregularis*) has adapted to show more diurnal and terrestrial habits in response to bird prey since its introduction in Guam [64]. Such adaptations have resulted in the extinction of several bird species and triggered ecosystem-level alterations [65]. Our results show that mink, after less than 20 years inhabiting Navarino Island as a new top predator, are showing more diurnal habits and occupying more terrestrial habitats than in the native range with potentially similar devastating effects on the local biota. Future research should further investigate our hypothesis of niche expansion at the time that mink management to control the invasion is urged, as the niche expansion may enhance the negative impacts of this predator on a myriad of small native vertebrates of the region.

## Supporting information

S1 Table Model selection results for American mink (*Neovison vison*) occupancy dynamics on Navarino Island, Chile for summer and spring 2014, and summer 2015. (PDF)

S1 Fig Model probability of detection (95% credible interval) as a function of distance to water (marine coast and freshwater) and ground cover for the best-supported dynamic occupancy models for the American mink on Navarino Island, Chile. (PDF)

S2 Fig Model coefficients and 95% credible intervals for the posterior distribution of the most parsimonious multi-season occupancy model for the American mink on Navarino Island for three seasons: Summer and spring 2014, and summer 2015. (PDF)

S3 Fig Model coefficients and 95% credible intervals for the posterior distribution for the single-season occupancy models for the American mink on Navarino Island for four consecutive seasons: Summer, winter, and spring 2014, and summer 2015. (PDF)

S1 Appendix Probability of detection of American mink as a function of days. (PDF)
