# Supplementary material for: Potential niche expansion of the American mink invading a remote island free of native-predatory mammals
Source: PLoS One. 2018 Apr 4;13(4):e0194745. doi: 10.1371/journal.pone.0194745 (PMC5884534; doi:10.1371/journal.pone.0194745)
Supplement: S1 Table — (PDF) [file pone.0194745.s001.pdf]

**S1 Table. Model selection results for American mink (*Neovison vison*) occupancy dynamics on Navarino Island, Chile for summer and spring 2014, and summer 2015.** Models in bold font indicate the model selected at each modelling stage. ΔDIC indicates the difference in DIC with respect to the null model. The model in bold font at the last stage is the model used for interpretation of the manuscript.

| Model | DIC | ΔDIC |
|---|---|---|
| **Models of detection probability (*p*)** | | |
| Null model | 862.6 | 0.0 |
| Distance to water | 849.9 | 12.7 |
| Ground cover | 846.0 | 16.6 |
| **Distance to water + Ground cover** | **851.7** | **10.9** |
| **Models of probability of occupancy for the first season ($\Psi_1$)** | | |
| Null model | 851.7 | 0.0 |
| **Altitude** | **833.5** | **18.2** |
| Slope | 861.5 | -9.8 |
| Distance to water | 894.0 | -42.3 |
| Habitat type | 871.6 | -19.9 |
| **Model of probability of colonization ($\gamma$)** | | |
| Null model | 833.5 | 0.0 |
| Autocorrelation | 852.3 | -18.8 |
| Distance to coast | 861.5 | -28.0 |
| Distance to coast^2 | 909.8 | -76.3 |
| Distance to water | 867.4 | -34.2 |
| **Distance to water^2** | **799.3** | **34.2** |
| Habitat type | 885.7 | -52.2 |
| **Model of probability of extinction (*e*)** | | |
| Null model | 799.3 | 0.0 |
| Autocorrelation | 813.8 | -14.5 |
| Distance to coast | 800.9 | -1.6 |
| Distance to coast^2 | 807.8 | -8.5 |
| **Distance to water** | **787.1** | **12.2** |
| Distance to water^2 | 791.3 | 8.0 |
| Habitat type | 792.3 | 7.0 |
| Distance to water + habitat type | 795.3 | 4.0 |
| Distance to water^2 + habitat type | 798.8 | 0.5 |
