# Supplementary material for: Potential niche expansion of the American mink invading a remote island free of native-predatory mammals
Source: PLoS One. 2018 Apr 4;13(4):e0194745. doi: 10.1371/journal.pone.0194745 (PMC5884534; doi:10.1371/journal.pone.0194745)

S1 Fig. Model probability of detection (95% credible interval) as a function of distance to water (marine coast and freshwater) and ground cover for the best-supported dynamic occupancy models for the American mink on Navarino Island, Chile.

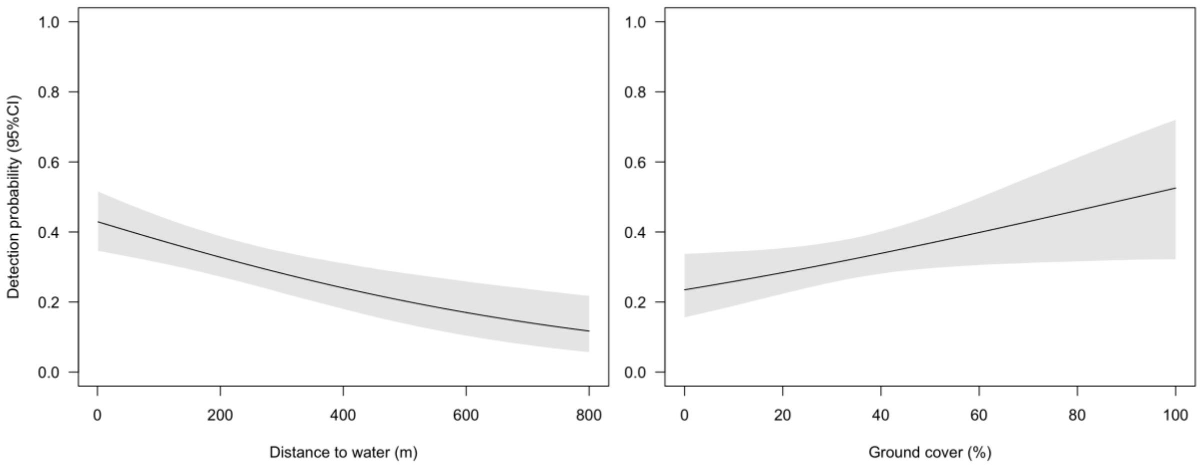

Supplement: S1 Fig — (PDF) [file pone.0194745.s002.pdf]
