# Supplementary material for: Potential niche expansion of the American mink invading a remote island free of native-predatory mammals
Source: PLoS One. 2018 Apr 4;13(4):e0194745. doi: 10.1371/journal.pone.0194745 (PMC5884534; doi:10.1371/journal.pone.0194745)

S2 Fig. Model coefficients and 95% credible intervals for the posterior distribution of the most parsimonious multi-season occupancy model for the American mink on Navarino Island for three seasons: summer and spring 2014, and summer 2015. Covariates include elevation, distance to water (marine coast and freshwater), and percentage of ground cover. Colonization and extinction parameters for Spring refer to the probability of colonization and extinction of sites from summer to spring. Colonization and extinction parameters for Summer refer to the probability of colonization and extinction of sites from spring to summer.

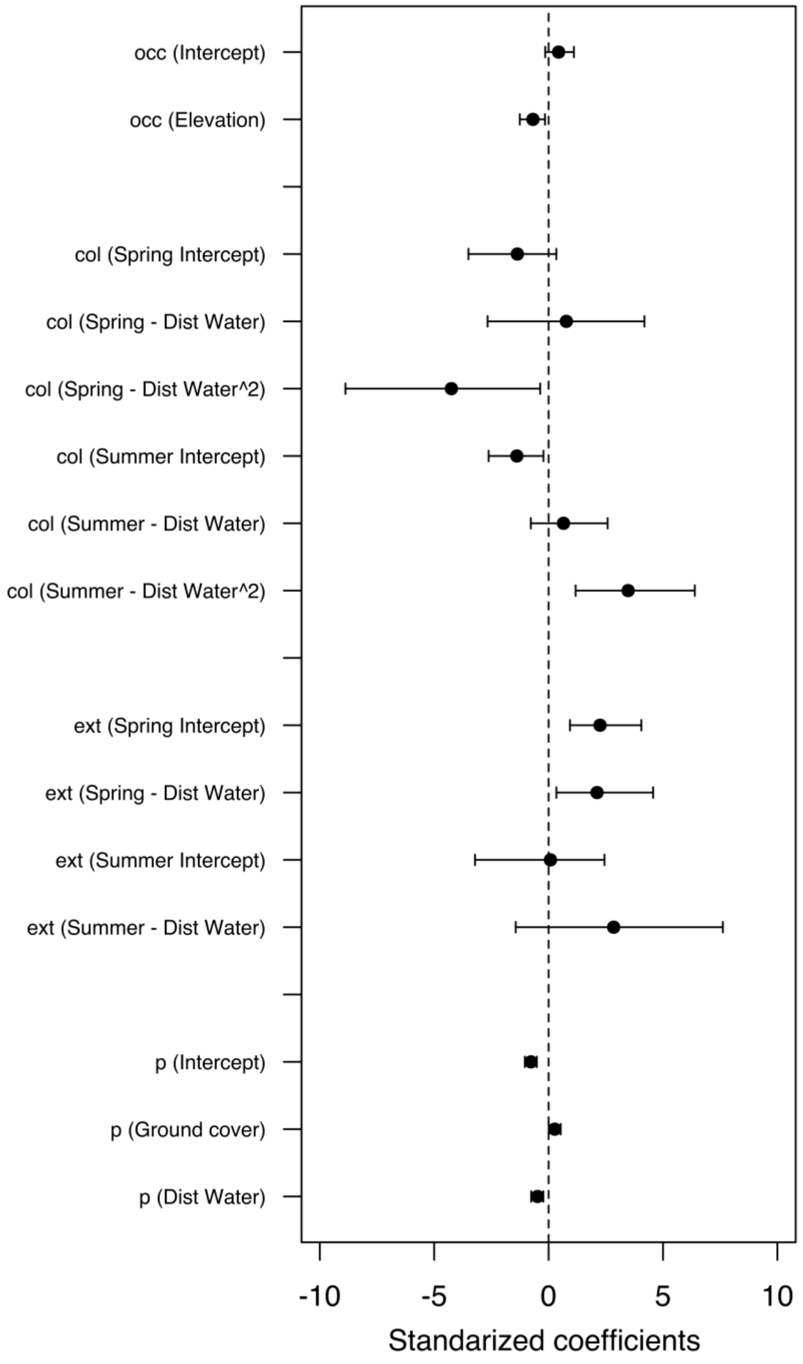

Supplement: S2 Fig — (PDF) [file pone.0194745.s003.pdf]
