# Supplementary material for: Potential niche expansion of the American mink invading a remote island free of native-predatory mammals
Source: PLoS One. 2018 Apr 4;13(4):e0194745. doi: 10.1371/journal.pone.0194745 (PMC5884534; doi:10.1371/journal.pone.0194745)

S3 Fig. Model coefficients and 95% credible intervals for the posterior distribution for the single-season occupancy models for the American mink on Navarino Island for four consecutive seasons: summer, winter, and spring 2014 and summer 2015. Covariates include distance to fresh water, and percentage of ground cover.

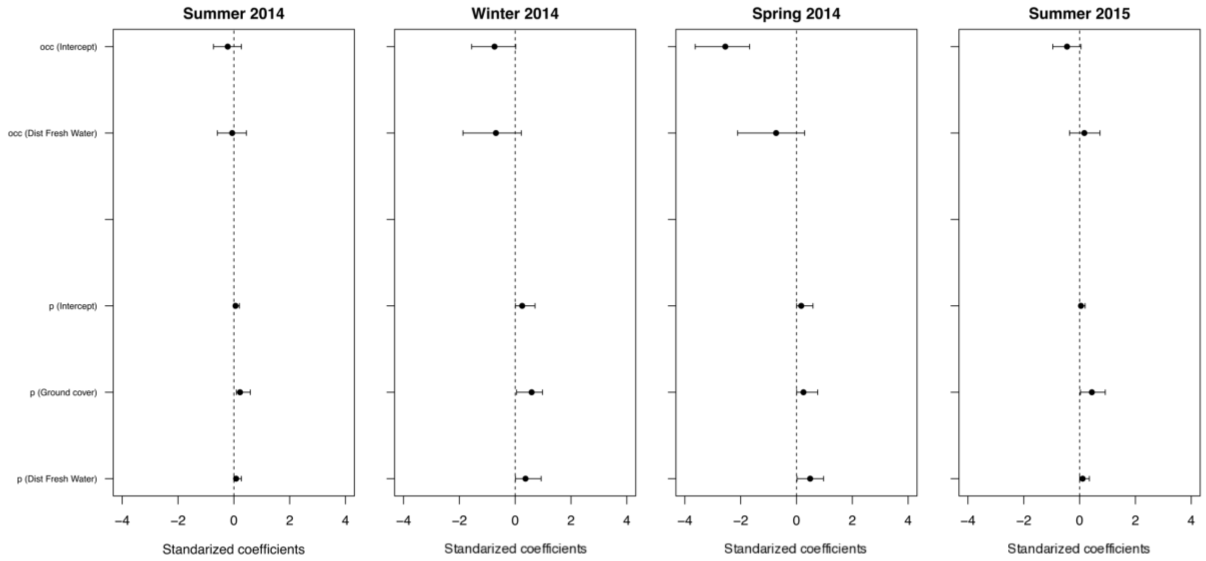

Supplement: S3 Fig — (PDF) [file pone.0194745.s004.pdf]
