# Supplementary material for: Potential niche expansion of the American mink invading a remote island free of native-predatory mammals
Source: PLoS One. 2018 Apr 4;13(4):e0194745. doi: 10.1371/journal.pone.0194745 (PMC5884534; doi:10.1371/journal.pone.0194745)
Supplement: S1 Appendix — (PDF) [file pone.0194745.s005.pdf]

S1 Appendix. Probability of detection of American mink as a function of days.

To ensure that mink will pass through the detection zone of the camera trap and to increase probability of detection we used bait [1]. Each camera was baited with can fish, which was placed inside a perforated can, and the can was attach to the floor making impossible for a mink or scavengers to remove neither the bait nor the can.

To assess whether bait influenced detection probability across the 20 days in which cameras were operative, we first created a detection history of detections and non-detections for the 20 days in which cameras were operative. We then modelled the probability of detection as a function of days by fitting a hierarchical multi-season occupancy model within a Bayesian framework [2]. We used the logit-link function to incorporate the covariate days in the model [2].

If bait has a strong effect on attracting animals the first days and then it losses the smell or disappears, we will expect detection probability to be negatively related to days. Otherwise, if the effect of bait remains constant, as we anticipated by using a can, we will expect detection probability to remain constant with days.

We implemented models using program JAGS [3], through package R2jags in R programing language [4]. We used 3 chains of Markov chain Monte Carlo (MCMC) to find 50,000 posterior distribution of the parameters of interest after a 5,000 burn-in period, using non-informative priors.

The posterior distribution of the beta parameter on detection probability confirmed that detection probability remained constant during the 20 days in which cameras were operative in this study (beta = -0.002 [95% credible intervals = -0.016 to 0.011]) (Fig S1). We concluded that the bait remained functional during the 20 days of sampling period. We then conducted multi-season occupancy model analysis without including bite as an effect on detection probability.

Fig S1. Probability of detection (95% credible intervals) of American mink per day for the 20 days' period in which cameras were active on Navarino Island, Chile.

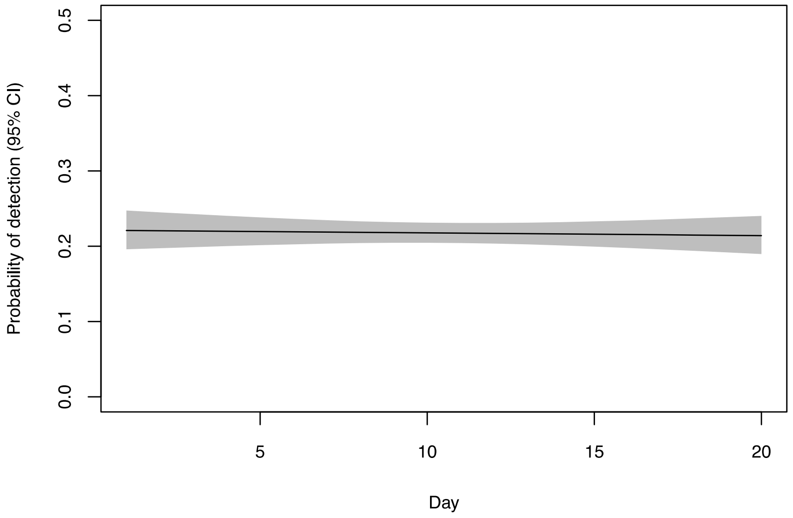

References

1. Gerber BD, Karpanty SM, Kelly MJ. Evaluating the potential biases in carnivore capture–recapture studies associated with the use of lure and varying density estimation techniques using photographic-sampling data of the Malagasy civet. Popul Ecol. 2012;54: 43–54. Available: http://link.springer.com/article/10.1007/s10144-011-0276-3
2. Royle JA, Dorazio RM. Hierarchical modeling and inference in ecology: the analysis of data from populations, metapopulations and communities. San Diego, California, USA: Academic Press; 2008.
3. Plummer M. JAGS: a program for analysis of Bayesian graphical models using Gibbs sampling. (Version 4.2). [Internet]. 2016. Available: http://mcmc-jags.sourceforge.net/
4. R Development Core Team. R: A language and environment for statistical computing. R Foundation for Statistical Computing, Vienna, Austria. URL https://www.R-project.org/. Vienna, Austria: R Foundation for Statistical Computing; 2016.
